# Effects of Novelty and Temporal Distance on Postexperience Spike Patterns of Hippocampal Place Cells Encoding Multiple Environments

**DOI:** 10.1523/JNEUROSCI.1639-24.2025

**Published:** 2025-11-25

**Authors:** Haruya Yagishita, Taiki Yokoi, Yu Shikano, Takuma Sato, Yuji Ikegaya, Takuya Sasaki

**Affiliations:** ^1^Department of Pharmacology, Graduate School of Pharmaceutical Sciences, Tohoku University, Sendai 980-8578, Japan; ^2^Laboratory of Chemical Pharmacology, Graduate School of Pharmaceutical Sciences, The University of Tokyo, Bunkyo-ku 113-0033, Japan; ^3^Center for Information and Neural Networks, Suita 565-0871, Japan; ^4^Institute for AI and Beyond, The University of Tokyo, Bunkyo-ku 113-0033, Japan; ^5^Department of Neuropharmacology, Graduate School of Medicine, Tohoku University, Sendai 980-8575, Japan

**Keywords:** CA3, hippocampus, multiple places, place cell, reactivation, sharp-wave ripple

## Abstract

The hippocampus plays a crucial role in consolidating episodic memories from diverse experiences that encompass spatial, temporal, and novel information. This study analyzed the spike patterns of hippocampal place cells in the CA3 and CA1 areas of male rats that sequentially foraged in five rooms, including familiar and novel rooms, followed by a rest period. Across the five rooms, both CA3 and CA1 place cells showed overlapping spatial representations. In a postexperience rest period, both CA3 and CA1 place cells increased baseline spike rates depending on the temporal distance from when the cells had place fields. In addition, CA3 place cells that encoded novel environments showed stronger sharp-wave ripple (SWR) reactivation. Coordinated reactivation of CA1 place cell ensembles that encoded temporally distant environments was eliminated. These results suggest that, following sequential experiences in multiple environments, increases in SWR-induced spikes of hippocampal neurons more specifically process novelty-related aspects of memory, while global increases in baseline spike rates process temporal distance-related aspects.

## Significance Statement

This study investigated how the hippocampus processes and stores memories from a series of experiences in different environments. While rats experienced familiar and novel rooms, both CA3 and CA1 neurons exhibited overlapping maps. In a postexperience rest period, these place cells increased baseline spike rates depending on the temporal distance from when the cells had place fields, suggesting processing of temporal distance-related aspects of memory. In addition, CA3 place cells that encoded novel environments specifically showed stronger reactivation during sharp-wave ripples, suggesting processing of novelty-related aspects. These differential activation patterns reveal how the hippocampus integrates spatial, temporal, and novelty information from multiple experiences.

## Introduction

From sequential experiences, animals need to extract and process information related to the temporal, spatial, and novel aspects of their experiences. The hippocampus plays a crucial role in episodic memory (Scoville and Milner, 1957; Squire et al., 2004) and contains neurons known as place cells that represent spatial information by firing at particular locations in an environment (O'Keefe and Dostrovsky, 1971; O'Keefe and Nadel, 1978). In different environments, spatial encoding patterns of place cells have been shown to differ between CA3 with recurrent networks and CA1 with feed-forward networks (Leutgeb et al., 2004, 2005). In CA3, distinct sets of hippocampal place cells independently encode spaces in different environments (Leutgeb et al., 2004, 2007; Alme et al., 2014), enabling orthogonalized spatial representations to minimize the interference of spatial maps by place cells. In CA1, place cells more frequently exhibit spatial representations within an environment, and a larger proportion of neurons consequently exhibit spatial representations across multiple environments compared with those in CA3 (Leutgeb et al., 2004, 2005). From a temporal perspective, the spatial firing patterns of CA3 neurons are more stable over time than those of CA1 neurons (Mankin et al., 2012; Sheintuch et al., 2023). The distinct stability of spatial encoding between the two subregions may underlie their unique roles in processing temporal information, such as temporal distance from experiences.

During rest/sleep periods, following the experience of an environment, place cell ensembles are synchronously reactivated, typically during sharp-wave ripples (SWRs) in local field potential (LFP) signals (Wilson and McNaughton, 1994; Skaggs and McNaughton, 1996; Kudrimoti et al., 1999; Csicsvari et al., 2000; O'Neill et al., 2008; Girardeau et al., 2009; Dupret et al., 2010; Buzsaki, 2015; van de Ven et al., 2016; Giri et al., 2019; Gridchyn et al., 2020; Nishimura et al., 2021; Yagi et al., 2023)_ENREF_12, which are believed to serve as neurophysiological substrates for memory consolidation. SWRs originate from the CA3 recurrent circuit, involving the reactivation of experienced information (Ecker et al., 2022), and subsequently establish coordinated neuronal spike assemblies in the downstream CA1 area (Buzsaki, 1989; Nakashiba et al., 2009). Crucially, both CA3 and CA1 place cells that encode novel than familiar environments exhibit more substantial increases in firing rates and firing associations during SWRs (Cheng and Frank, 2008; O'Neill et al., 2008; Giri et al., 2019; Hwaun and Colgin, 2019), potentially offering an efficient mechanism for integrating novel information into hippocampal circuits.

The significance of hippocampal spike patterns raises more intricate questions regarding how reactivation occurs when place cells encode multiple environments consisting of a combination of various spatial, temporal, and novel/familiar information. To clarify these issues, we recorded the spike patterns of hippocampal CA3 and CA1 place cells as rats sequentially experienced five different rooms, alternating between familiar and novel rooms, and then rested in the familiar box. After confirming the spatial representation patterns across multiple rooms by CA3 and CA1 place cells, we analyzed how postexperience activation of the place cell ensembles was affected by the novelty of the environments and temporal distance from the experiences.

## Materials and Methods

### Experimental model and subject details

All experiments were performed with the approval of the experimental animal ethics committee at the University of Tokyo (approval number, P29-7) and the Committee on Animal Experiments at Tohoku University (approval number, 2022 PhA-004) and according to the NIH guidelines for the care and use of animals.

Male Long–Evans rats (3–6 months old) with a preoperative weight of 400–500 g were purchased from SLC and used in this study. The rats were housed individually and maintained on a 12 h light/12 h dark schedule with lights off at 7:00 A.M. Following at least 1 week of adaptation to the laboratory, the rats were reduced to 85% of their *ad libitum* weight through limited daily feeding. Water was readily available.

### Behavioral habituation to familiar fields before surgery

All behavioral experiments were conducted during the dark phase. The experiments were conducted in darkened rooms illuminated by a single overhead light source (10 cm diameter) mounted on the wall that could emit different colors (blue, yellow, pink, purple, red, white, green, or orange; Fig. S1). The light intensity in the behavioral arena was maintained at 10 lux across all color conditions. Before surgery, rats were trained to perform random foraging in three different rooms (Rooms 1, 3, and 5). Each room was positioned at a different location and orientation, all separated by black curtains with different global cues. Room 1 was a 1 m square with a gray foam floor and blue lighting. Room 3 had a hexagonal shape with two 90° angles and four 135° angles, situated within a 1 m square area with a blue plastic floor and pink lighting. Room 5 was in the form of a trapezoid with an upper base of 0.5 m, a lower base of 1 m, and a height of 1 m, featuring a blue plastic floor and green lighting. The structures of Room 2 and Room 4 were described in the Legend of Figure S1. All rooms had a wall height of 20 cm and were elevated 50 cm above the floor.

On the training day, the rats first rested in a rest box (30 × 30 cm) outside the rooms for 30 min and then sequentially experienced the three rooms (Rooms 1, 3, and 5). In each session, the rats were placed in a room and habituated to the environment by allowing them to freely forage for randomly placed chocolate milk samples for 10 min. Between sessions, they returned to the same rest box for 5 min. After three sessions, the rats rested in the same box for 30 min. This training was repeated daily until the rats consumed the reward at least 20 times in a 10 min session. To achieve this criterion, the training lasted for at least 4 d.

### Surgical procedures

The rat was anesthetized with isoflurane gas (0.5–2.5%) and secured on a flat heat pad. Veterinary ointment was placed on the eyes to prevent dryness during anesthesia. Buprenorphine (0.05 mg/kg, s.c.) was administrated as an analgesic. A 2 cm midline incision was made in the area between the eyes and the cerebellum. A craniotomy with a diameter of 1.5 mm was created above the right dorsal hippocampus (3.5 mm posterior and 3.3 mm lateral to the bregma) using a high-speed drill, and the dura was surgically removed. Two stainless steel screws were implanted in the bone above the prefrontal cortex to serve as ground electrodes. An electrode assembly consisting of 13–15 independently movable tetrodes was stereotaxically implanted above the craniotomy (Okada et al., 2017; Aoki et al., 2019; Nishimura et al., 2021). The tips of the tetrode bundles were lowered to the cortical surface, and the tetrodes were inserted 1.0 mm into the brain at the end of surgery. The tetrodes were constructed from 17-μm-wide polyimide-coated platinum–iridium (90/10%) wire (California Fine Wire California Fine Wire) and plated with platinum to reduce their electrode impedances to 150–300 kΩ at 1 kHz. All recording devices were secured to the skull using stainless steel screws and dental cement. Following surgery, each rat was housed individually in a transparent Plexiglass with *ad libitum* access to water and food for at least 5 d. After recovery from the surgery, they were then food-deprived until they reached 85% of their previous body weight.

### Adjusting electrode depth

The rat was connected to the recording equipment via Cereplex M (Blackrock), a digitally programmable amplifier, close to the rat's head. The output of the headstage was conducted via a lightweight multiwire tether and a commutator to the Cereplex Direct recording system (Blackrock), a data acquisition system. Electrode turning was performed while the rat was resting in a rest box. Over a period of at least 2 weeks after surgery, tetrode tips were advanced slowly 25–100 μm per day for 14–21 d until spiking cells were encountered in the CA1 layer of the hippocampus, which was identified on the basis of LFP signals and single-unit spike patterns. Once the tetrodes were adjacent to the CA1 cell layer, as indicated by the presence of low-amplitude multiunit activity, tetrodes were settled for stable recordings over a period of several days. After recordings from CA1 cells, the tetrodes were further advanced slowly 25–100 μm per day for 14–21 d until spiking cells were encountered in the CA3 layer of the hippocampus, which was identified on the basis of LFP signals and single-unit spike patterns. Once the tetrodes were adjacent to the CA3 cell layer, recordings were obtained over a period of several days.

### Recording paradigm

After surgery, behavioral habituation resumed at least 5 d before the recording day. Electrophysiological recordings were conducted after the rats consumed the reward at least 20 times within the 10 min session for at least 3 consecutive days. On the recording day, the rats initially rested in the box for 30–60 min (prerest) and then sequentially explored five rooms (Rooms 1, 2, 3, 4, and 5). Finally, the rats rested in a rest box for 30–60 min (postrest). Similar to the training, the rats freely foraged in each chamber for 10 min and rested for 5 min between sessions, during which the floor of the field was cleaned with water and 70% ethanol. Rooms 2 and 4 were novel, with trapezoidal and square shapes, respectively (except for Rat 1). Every day, these novel rooms featured distinct flooring materials such as polyurethane, plastic, corrugated board, or artificial turf, along with wall cues provided by green tape, black tape, a black star, or red and yellow squares (for more details, see Fig. S1). The walls were either blue or white, and the lighting was in various colors, including yellow, orange, purple, red, and white. The rooms had wall heights of 30 or 60 cm and were elevated 50 cm above the floor. All rooms were positioned in different locations and orientations and were separated by black curtains with different global cues (e.g., room poster and walls).

### Electrophysiological data collection

Electrophysiological data collection commenced after stable well-separated unit activity was identified in the hippocampus and the rat reached the criterion performance. LFP recordings were sampled at 2 kHz and low-pass filtered at 500 Hz. Unit activity was amplified and high-pass filtered at 750 Hz. Spike waveforms above a trigger threshold (50 μV) were timestamped and recorded at 30 kHz for 1.6 ms. To monitor the rat's moment-to-moment position, we attached an infrared light reflective tape to the microdrive, and the LED signal position was tracked at 25 Hz using an infrared camera located on the ceiling and sampled by a laptop computer. All the recording equipment placed between the recording room and the room entrance was wheeled across rooms without stopping the recordings to ensure identical recording conditions (e.g., sampling, filtering, and amplification). Recordings were conducted for at least 2 d.

### Histological analysis to confirm electrode placement

After the experiments, the rats received an overdose of urethane and were intracardially perfused with 4% paraformaldehyde (PFA) in phosphate-buffered saline (PBS) and decapitated. To aid in the reconstruction of the electrode tracks, the electrodes were not withdrawn from the brains until more than 3–4 h after perfusion. After dissection, the brains were fixed overnight in 4% PFA and then equilibrated with 30% sucrose in PBS. Frozen coronal slices (50 μm) were cut using a microtome, and serial sections were mounted. The slices were rinsed in water, counterstained with cresyl violet, and coverslipped with hydrophobic mounting medium. The positions of all the tetrodes were confirmed by identifying the corresponding electrode tracks in the histological tissue with an optical microscope.

### Spike sorting

Spike sorting was performed offline using the graphical cluster-cutting software MClust. Rest recordings before and after the behavioral paradigms were included in the analysis to assure recording stability throughout the experiment and to identify hippocampal cells that were silent during behavior. Clustering was performed manually in 2D projections of the multidimensional parameter space (i.e., comparisons between waveform amplitudes, the peak-to-trough amplitude differences, and waveform energies, each measured on the four channels of each tetrode). The cluster quality was measured by computing the *L*_ratio_ and isolation distance. A cluster was considered as a cell when the *L*_ratio_ was <0.38 (average *L*_ratio_ was 0.091 ± 0.004 in 619 isolated cells). In the autocorrelation histograms, cells with no clear refractory period (<3 ms) were excluded from analyses. Refractory periods of spikes were considered to increase confidence in the successful isolation of cells. In addition, in the cross-correlation histograms, putative cell pairs with a symmetrical gap around the center bins were considered to arise from the same cell and were merged.

### Analysis of spatial spike patterns

The following analyses were applied for each cell. For quantifying spatial selectivity, a spatial firing-rate distribution was constructed by dividing the sum of the total number of spikes in each location bin (7 × 7 cm) by the amount of time that the animal spent in that bin. Data were smoothed with a Gaussian kernel filter with a standard deviation of 1.5 pixel (11 cm), constructing a tuning curve map. A spatial information density was computed by the following formula:

$$I=\overset{N}{\underset{i\;=\;1}{\sum }}\;p_{i}\frac{r_{i}}{r}\log_{2}\frac{r_{i}}{r},$$

where *I* is the spatial information density measured in bits per spike, *i* is the index of the pixels of the place field, *p_i_* is the probability of the animal being at location *i*, *r_i_* is the average firing rate of the cell when the animal is at location *i*, and *r* is the total average firing rate. A cell was classified as a place cell in each room if the following criteria were met: (1) the spatial information was >0, (2) the spatial information was >1% of the top of those computed from 1,000 randomized data in which spike times were shifted along the recording period by a random amount within the duration of the recording session, and (3) the average firing rate in the room was >0.05 Hz, and (4) the maximum firing rate among all bins was >0.5 Hz.

**Figure 1.**
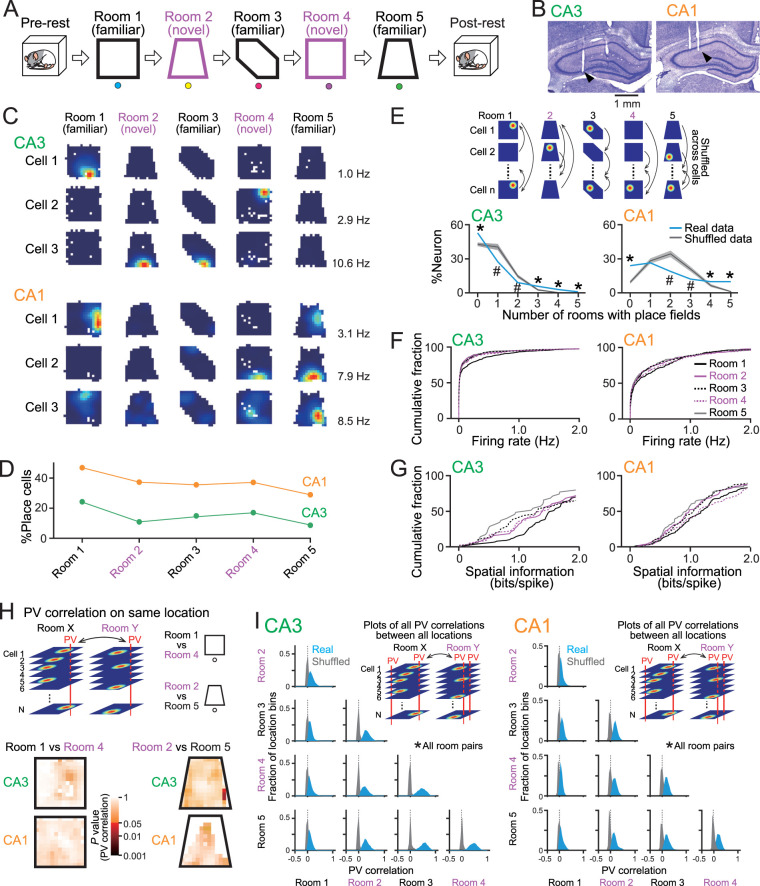
Spatial encoding of hippocampal neurons in the five rooms. ***A***, On the day of recording, a rat sequentially experienced three familiar and two novel rooms. Before and after the sessions, the rat rested in a box, termed prerest and postrest periods. All room patterns are summarized in Figure S1. ***B***, Representative images of cresyl violet-stained brain sections showing tetrode locations in the CA3 and CA1 (arrowhead). The numbers of cells in all rats are summarized in Figure S1. All electrode locations are summarized in Figure S2. Spike sorting results are presented in Figure S3. ***C***, Color-coded firing–rate maps of representative CA3 and CA1 cells in the five rooms (blue, 0 Hz; red, peak rate indicated by the left bottom in each panel). ***D***, The percentages of place cells identified in each room (CA3, *n* = 352 cells; CA1, *n* = 267 cells). ***E***, The probability distributions of the number of rooms in which place fields were observed from single neurons. Real and shuffled data are shown in black and cyan, respectively. Data are presented as the mean ± SD. *p < 0.05 (real larger) and ^#^*p* < 0.05 (shuffle larger), Student's *t* test. ***F***, Cumulative distributions of average firing rates in each room (CA3, *n* = 352 cells; CA1, *n* = 267 cells). ***G***, Cumulative distributions of spatial information of place cells identified in each room (CA3, *n* = 89, 38, 52, 59, and 35 place cells; CA1, *n* = 129, 100, 96, 100, and 78 place cells in Rooms 1, 2, 3, 4, and 5, respectively). ***H***, Top, Correlation coefficients of PVs were computed for corresponding location bins for pairs of rooms with identical geometry [Room 1 vs Room 4 (square) and Room 2 vs Room 5 (trapezoid)]. Bottom, Heatmaps showing statistical *p* values of PV correlation coefficients computed between real and shuffled datasets (as described in Fig. 1*E*). The distributions were constructed from all rats. ***I***, For each pair of rooms, distribuetions of PV correlations were constructed between all pairs of locations (left, CA3; right, CA1). Distributions were significantly different between real (blue) and shuffled (gray) datasets in all room pairs (*p* < 0.01, Mann–Whitney *U* test followed by Bonferroni’s correction).

To assess whether the observed distribution of place fields across rooms in single-place cells could occur by chance, we created shuffled datasets in which spatial maps of each neuron were randomly reassigned to different neurons while maintaining the total number of place cells per room (Fig. 1*E*, top). This shuffling procedure eliminated any systematic biases in how individual neurons represented space across multiple rooms. The shuffling procedure was repeated 1,000 times.

A population vector (PV) was constructed from the spatial firing-rate distributions of all the recorded place cells. Correlation coefficients for all pairs of PVs were computed to construct a PV correlation matrix. PV correlations from real datasets were compared with those observed from shuffled datasets as described above.

### Detection of synchronous events and SWRs

The electrode including the largest number of putative pyramidal cells identified in the spike sorting process was used for SWR detection. LFP signals during rest periods were bandpass filtered at 150–250 Hz, and the root mean square was calculated with a bin size of 10 ms. SWR events were detected if the power exceeded a threshold for at least 20 ms. The threshold for SWR detection was set to 3 standard deviations (SDs) above the mean of all envelopes computed from the rest periods. SWR events that occurred within 100 ms from the former SWR events were excluded. The onset of SWRs was marked at the point when the root mean square first exceeded 3 SDs above the mean. SWR-associated spike rates were computed from −50 to +50 ms relative to SWR onsets. For each cell, a SWR-induced change ratio was computed as the ratio of the difference in its SWR-associated spike rates between the prerest and postrest periods to the sum of these rates. The ratios ranged between −1 and +1.

### Linear regression of rate change ratios and SWR-induced change ratios

For the *l*th place cell, a (5 + 1)-dimensional predictor vector $X_{l}$ consists of five-dimensional entries, each representing a binary index indicating the presence (1) or absence (0) of its place fields in each room. The (5 + 1)th entry was set to +1 as a constant term. The dependent variable $Y_{l}$ was set as the SWR-induced change ratio. A linear relationship between a series of predictor vectors, $X=(X_{1},\;X_{2},\;X_{3},\cdot \cdot \cdot ,X_{L}{)}^{T}$, and a series of dependent variables, $Y=(Y_{1},\;Y_{2},\;Y_{3},\cdot \cdot \cdot ,Y_{L}{)}^{T}$, where *L* denotes the total number of place cells, was estimated by a linear regression analysis in which the best (5 + 1)-dimensional weighted coefficients *β* were mathematically computed so that the weighted linear sum *Y*’ (=*X · β*) was fitted against *Y* as follows:

$$\beta =X^{+}Y,$$

where $X^{+}=(X^{T}X{)}^{-1}X^{T}$.

The effects of novelty, familiarity, and temporal distance on rate change ratios and SWR-induced change ratios were estimated using multiple linear regressions. For the *l*th place cell, three predictor vectors were created. The first is a (5 + 1)-dimensional predictor vector representing novelty $X\_\mathrm{nove}l_{l}$ which is the product of $X_{l}$ and a constant vector novel = (0, 1, 0, 1, 0, 1), composed of five-dimensional entries, each representing the binary index indicating the novel (1) or familiar (0) rooms. The (5 + 1)th entry was set to +1 as a constant term. The second is a similar predictor vector representing familiarity $X\_\mathrm{familiarit}y_{l}$ which is the product of $X_{l}$ and a constant vector familiarity = (1, 0, 1, 0, 1, 1). The third is a (5 + 1)-dimensional predictor vector representing the decay effects of the temporal distance $X\_\mathrm{tem}p_{l}$ which is the product of $X_{l}$ and a constant vector Temp, composed of five-dimensional entries of a decay function over time, assuming that the effects of reactivation undergo linear decay, and the (5 + 1)th entry of +1 as a constant term. To account for the unknown decay time constant, we systematically varied the decay constant Δ across different attenuation rates, where higher Δ values modeled stronger temporal decay from earlier rooms. The temporal vector was constructed as follows: when Δ = 0, Temp = (1, 1, 1, 1, 1, 1); when Δ = 0.2, Temp = (0.2, 0.4, 0.6, 0.8, 1, 1); when Δ = 0.4, Temp = (0, 0, 0.2, 0.6, 1, 1); and when Δ = 1, Temp = (0, 0, 0, 0, 1, 1). A linear relationship between three predictor vectors, X_novel, X_familiarity, and X_temp, and the dependent variables Y was estimated by a multiple linear regression analysis in which the best (5 + 1)-dimensional weighted coefficients *β*_novel_, *β*_fam_, and *β*_temp_ were mathematically computed so that the weighted linear sum *Y'* (=*X*_novel · *β*_novel_ + *X*_familiarity · *β*_fam_ + *X*_temp · *β*_temp_) was fitted against *Y*. The *β* values were computed including the 95% confidence interval. The validity of the linear regression model was quantified by *F* values and *p* values.

### Joint spike probability and cofiring

To measure the degree to which a given cell pair exhibited significant joint spikes during rest periods, we converted spike patterns in each neuron to a *N*_pre_- and *N*_post_-dimensional vector containing 1 or 0 depending on whether the neuron showed spikes or not in a 100 ms bins in prerest and postrest, respectively, where *N*_pre_ and *N*_post_ denote the total numbers of 100 ms bins (typically 18,000 bins from 30 min). For each neuronal pair, the joint spike probability was calculated as the ratio of the number of bins where both neurons showed spikes (entries in both vectors were 1) to the total number of bins, termed Prob_pre_ and Prob_post_. A change in joint spike probabilities from the pre- to the postrest period was quantified by a probability change ratio as follows:

$$\mathrm{Probability}\;\mathrm{change}\;\;\mathrm{ratio}=\frac{\mathrm{Pro}b_{\mathrm{post}}-\mathrm{Pro}b_{\mathrm{pre}}}{\mathrm{Pro}b_{\mathrm{post}}+\mathrm{Pro}b_{\mathrm{pre}}}.$$

If both Prob_pre_ and Prob_post_ were 0, the ratio was not computed.

In addition, for each neuron pair, cofiring_room_ was computed in each room as follows:

$$\mathrm{Cofirin}g_{\mathrm{room}}=\frac{\sum_{i\;=\;1}^{N}\;(x_{i}-\bar{x})(y_{i}-\bar{y})}{\sqrt{\sum_{i\;=\;1}^{N}\;{\left(x_{i}-\bar{x}\right)}^{2}}\sqrt{\sum_{i\;=\;1}^{N}\;{\left(y_{i}-\bar{y}\right)}^{2}}},$$

where *N* was the total number of 100 ms bins in a room (e.g., 6,000 bins from 10 min), *x_i_* and *y_i_* were the number of spikes of cell *x* and *y*, respectively, at *i*th bin, and $\bar{x}$ and $\bar{y}$ were the average spike rates of each neuron in the room. Cells with no spikes in the room were excluded from this analysis. Cofiring pairs were defined as neuron pairs with cofiring_room_ of >0.03 (corresponding to *p* < 0.01; this criterion was determined from shuffled datasets of randomly generated spike patterns for neuron pairs at ∼0.5 Hz).

### Statistical analysis

All data were analyzed using the MATLAB software. Two-sample data were compared using Student's *t* test, Mann–Whitney *U* test, and Wilcoxon signed-rank test. Multiple group comparisons were performed using post hoc Bonferroni’s correction. The probabilities of observing place cells were compared using the *χ*^2^ test. The null hypothesis was rejected at the *p* < 0.05 level, unless otherwise specified. Data were presented as mean ± standard error of the mean (SEM), unless otherwise specified.

## Results

### Experimental timeline

Rats were trained daily for 1 week to forage for food rewards in three rooms (Rooms 1, 3, and 5) with different shapes, each placed in a different location surrounded by different external cues. Subsequently, these rooms were used as familiar rooms (Fig. 1*A*; Fig. S1). After the rats were implanted with the tetrode assembly, they were again trained daily in the three identical rooms, while the tetrodes were advanced to the hippocampal CA1 pyramidal cell layer. On the day of CA1 neuron recording, the rats first rested in a familiar box (prerest) and successively performed random foraging in five rooms from Room 1 to 5 every 10 min, each of which was flanked by a rest interval of 5 min in the same box. Finally, the rats rested again in the same box (postrest; Fig. 1*A*). Among these five rooms, Rooms 2 and 4 were novel for the rats, situated in different settings with distinct external cues compared with the three familiar rooms (Fig. S1). After recording CA1 neuronal spikes for several days, tetrodes were advanced into the hippocampal CA3 pyramidal cell layer in the same rats. Recordings were then obtained from the CA3 neurons using the same procedures, except that Rooms 2 and 4 were situated in completely different settings, including different cues and room coloring, so that they served as novel rooms every time. A limitation of our study design was that, as CA3 recordings followed CA1 recordings in five rats out of nine rats, these rats had greater familiarity with the environment during CA3 sessions. This sequential recording approach might have influenced some effects on CA1 and CA3 spike patterns.

### Spatial encoding by hippocampal CA3 and CA1 neurons in multiple rooms

In total, 352 and 267 putative excitatory pyramidal neurons were recorded from the CA3 and CA1 areas in 16 and 10 recordings, respectively, from a total of nine rats (Fig. 1*B*). The majority of CA3-targeted electrodes were positioned within the CA3b subregion (Fig. S2). The amplitudes of spike waveforms in active neurons were stable throughout a recording period, including the prerest and postrest periods (Fig. S3). Overall, the proportions of CA1 neurons showing place fields in individual rooms were higher than those of CA3 neurons (Fig. 1*C*,*D*). In both CA3 and CA1, no significant differences were observed in the proportions of neurons showing place fields in familiar or novel rooms (Fig. 1*D*; CA3, *χ*^2^ = 2.69; *p* = 0.10; CA1, *χ*^2^ = 0.019; *p* = 0.89; *χ*^2^ test). The number of rooms in which place fields were observed from single CA1 neurons was significantly higher than that from single CA3 neurons (Fig. 1*E*; CA3, *n* = 164 place cells; CA1, *n* = 205 place cells; *Z* = 9.41; *p* = 5.0 × 10^−21^; Mann–Whitney *U* test). To evaluate whether the number of rooms with place fields observed from single-place cells across the five rooms could be statistically explained by chance, we created shuffled datasets in which the spatial maps of individual neurons were randomly assigned to other neurons (Fig. 1*E*, top). In CA3, the proportion of neurons showing no place fields in any of the five rooms was significantly higher in the real datasets than in the shuffled datasets (Fig. 1*E*, left; *n* = 188 cells; *t*_(999)_ = 7.00; *p* = 4.8 × 10^−12^, Student's *t* test). On the other hand, the proportions of neurons showing one and two place fields out of the five rooms were significantly lower (1, *n* = 101 cells; *t*_(999)_ = 4.29; *p* = 4.8 × 10^−5^; 2, *n* = 31 cells; *t*_(999)_ = 4.02; *p* = 6.3 × 10^−5^), whereas those showing three, four, and five place fields were significantly higher, compared with shuffled datasets (3, *n* = 20 cells; *t*_(999)_ = 4.27; *p* = 2.1 × 10^−5^; 4, *n* = 10 cells; *t*_(999)_ = 10.62; *p* = 4.9 × 10^−25^; 5, *n* = 2 cells; *t*_(999)_ = 11.03; *p* = 9.4 × 10^−27^). In CA1, the proportion of neurons showing no place fields in any of the five rooms was significantly higher in the real datasets than in the shuffled datasets (Fig. 1*E*, right; *n* = 62 cells; *t*_(999)_ = 10.01; *p* = 1.6 × 10^−22^, Student's *t* test). On the other hand, the proportions of neurons showing two and three place fields out of the five rooms were significantly lower (2, *n* = 52 cells; *t*_(999)_ = 5.28; *p* = 1.6 × 10^−7^; 3, *n* = 32 cells; *t*_(999)_ = 4.09; *p* = 4.6 × 10^−5^), whereas those showing four and five place fields were significantly higher, compared with shuffled datasets (4, *n* = 26 cells; *t*_(999)_ = 2.85; *p* = 0.0044; 5, *n* = 26 cells; *t*_(999)_ = 17.78; *p* = 1.1 × 10^−61^). These results confirm that subsets of CA3 and CA1 neurons preferentially maintained no spatial selectivity or generated more frequent spatial representations across multiple environments than expected by chance. No significant differences in the average firing rates were observed among the five rooms (Fig. 1*F*; CA3, *n* = 352 cells; CA1, *n* = 267 cells; *p* > 0.05; Mann–Whitney *U* test followed by Bonferroni’s correction for all comparisons). Moreover, no significant differences in the spatial information of place fields were observed among the five rooms (Fig. 1*G*; CA3, *n* = 89, 38, 52, 59, and 35 place cells; CA1, *n* = 129, 100, 96, 100, and 78 place cells; *p* > 0.05, Mann–Whitney *U* test followed by Bonferroni’s correction in all comparisons), except Room 1 versus Room 5 in CA3 (*Z* = 3.06; *p* = 0.22).

To further confirm spatial representations across multiple environments at the neuronal ensemble level, a PV was constructed from the firing rates of all place cells within a 7 × 7 cm location bin in a spatial map of each room (Fig. 1*H*). First, we analyzed whether identical spatial representation patterns by neuronal ensembles were observed at the identical locations between room pairs with identical geometry [Room 1 vs Room 4 (square) and Room 2 vs Room 5 (trapezoid)]. For each pair of rooms, correlation coefficients of PVs were computed for corresponding location bins. Shuffled datasets were computed as described in Figure 1*E*, and statistical *p* values were determined by comparing real and 1,000 shuffled datasets using Student's *t* test. Both CA3 and CA1 cells showed very few significant bins among all location bins tested in both between Room 1 and Room 4 and between Room 2 and Room 5 (Fig. 1*H*, bottom). These results suggest that hippocampal place cell ensembles exhibit distinct spatial representations at corresponding spatial locations across different environments, even when those environments share identical geometric configurations. Next, we tested whether these neuronal populations show overlapping spatial representation patterns at any locations across different environments. PV correlations were computed from all possible pairs of location bins (Fig. 1*I*). In both CA3 and CA1 neurons, distributions of PV correlations were significantly skewed to the positive side compared with those computed from the corresponding 1,000 shuffled datasets (Fig. 1*I*; *p* < 0.01, Mann–Whitney *U* test followed by Bonferroni’s correction). These results demonstrate that the spatial representation patterns of CA3 and CA1 place cells exhibit some overlaps across different environments, even though these overlaps do not occur at corresponding location bins.

### Effects of the number of spatial representations on postexperience changes in overall spike rates of CA3 and CA1 place cells during rest periods

**Figure 2.**
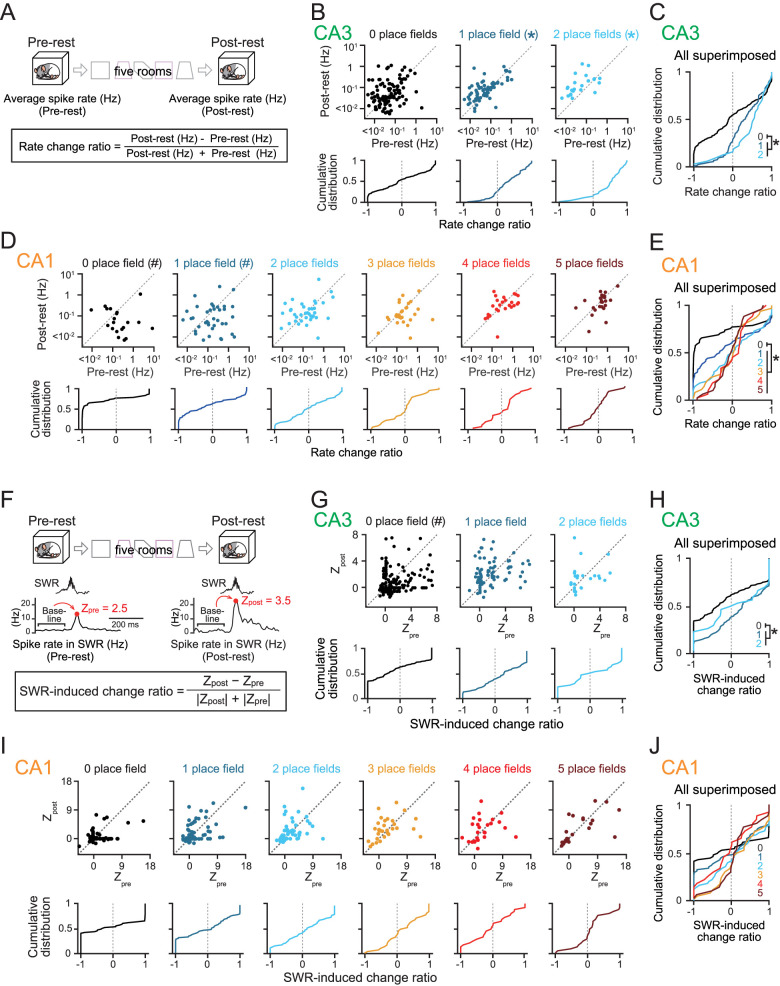
Postexperience reactivation of place cells showing different counts of spatial representations. ***A***, Spike rates during the entire pre- and postrest periods were compared. A rate change ratio was computed from each neuron. ***B***, Top, Scatterplots of overall spike rates of CA3 neurons during the postrest period against those during the prerest period, separately created for each number of rooms (0, *n* = 188 cells; *Z* = 0.93; *p* = 0.35; 1, *n* = 101 cells; *Z* = 4.02; *p = 5.7 × 10^−5^; 2, *n* = 31 cells; *Z* = 2.82; *p = 0.0048). *p < 0.05 (postlarger), Wilcoxon signed-rank test. Bottom, Cumulative distributions of rate change ratios computed from the scatterplots. ***C***, All distributions shown in ***B*** are superimposed (*n* = 188 and 164 cells; *Z* = 4.19; *p = 2.8 × 10^−5^; 0 vs 1–2, Mann–Whitney *U* test). ***D***, Same as ***B*** but for CA1 neurons (0, *n* = 62 cells; *Z* = 3.92; ^#^*p* = 9.0 × 10^−5^; 1, *n* = 69 cells; *Z* = 2.10; ^#^*p* = 0.035; 2, *n* = 52 cells; *Z* = 0.96; *p* = 0.34; 3, *n* = 32 cells; *Z* = 0.010; *p* = 0.99; 4, *n* = 26 cells; *Z* = 0.26; *p* = 0.79; 5, *n* = 26 cells; *Z* = 0.19; *p* = 0.85, Wilcoxon signed-rank test). ***E***, Same as ***C*** but for CA1 neurons (*n* = 62 and 205 cells; *Z* = 4.43; *p = 9.4 × 10^−6^; 0 vs 1–5, Mann–Whitney *U* test). ***F***, SWR-associated spike rate increases in the pre- and postrest periods were compared by computing a SWR-induced change ratio from each neuron. ***G***, Top, Scatterplots of *Z*_pre_ and *Z*_post_ of CA3 neurons, separately created for each number of rooms (0, *n* = 188 cells; *Z* = 3.12; ^#^*p* = 0.0018; 1, *n* = 101 cells; *Z* = 1.44; *p* = 0.15; 2, *n* = 31 cells; *Z* = 0.65; *p* = 0.52). ^#^*p* < 0.05 (prelarger), Wilcoxon signed-rank test. Bottom, Cumulative distributions of SWR-induced change ratios computed from the scatterplots. ***H***, All distributions shown in ***G*** are superimposed (*n* = 188 and 164 cells; *Z* = 3.97; *p = 7.3 × 10^−5^; 0 vs 1–2, Mann–Whitney *U* test). ***I***, Same as ***G*** but for CA1 neurons (0, *n* = 62 cells; *Z* = 0.19; *p* = 0.85; 1, *n* = 69 cells; *Z* = 0.019; *p* = 0.99; 2, *n* = 52 cells; *Z* = 0.60; *p* = 0.55; 3, *n* = 32 cells; *Z* = 1.68; *p* = 0.09; 4, *n* = 26 cells; *Z* = 0.040; *p* = 0.97; 5, *n* = 26 cells; *Z* = 1.00; *p* = 0.32, Wilcoxon signed-rank test). ***J***, Same as ***H*** but for CA1 neurons.

Next, we investigated how individual hippocampal cells were activated in the postrest period after the rats experienced the five rooms. As postexperience spike patterns of hippocampal cells have been shown to represent not only experience-dependent neuronal activity but also preconfigured neuronal population activity that emerges during pre-experience periods (Dragoi and Tonegawa, 2011; Grosmark and Buzsaki, 2016; Liu et al., 2018, 2019), we evaluated spike patterns in the postrest period, compared with those in the prerest period. First, we confirmed how overall spike rates of individual hippocampal neurons during the postrest period were altered from those during the prerest period by constructing scatter plots separately for different numbers of rooms in which individual neurons showed place fields (Fig. 2*A*). The spike rates of CA3 place cells that had place fields in at least one room during the postrest period were significantly higher than those during the prerest period (Fig. 2*B*,*C*; 1, *n* = 101 cells; *Z* = 4.02; *p* = 5.7 × 10^−5^; 2, *n* = 31 cells; *Z* = 2.82; *p* = 0.0048, Wilcoxon signed-rank test). In contrast, CA3 neurons with no place fields showed no significant differences in spike rates between the prerest and postrest period (Fig. 2*B*; CA3; 0, *n* = 188 cells; *Z* = 0.93; *p* = 0.35). In addition to spike rate changes, a rate change ratio was computed as the ratio of the difference in overall spike rates between the entire pre- and postrest periods to the sum of these rates (Fig. 2*A*). The ratios ranged between −1 and +1, with higher positive and negative ratios indicating a stronger bias toward the prerest and postrest periods, respectively. Cells that generated no spikes in both the pre- and postrest periods were excluded from the analysis. The rate change ratios of CA3 neurons that had place fields in at least one room were significantly higher than those that had no place fields (Fig. 2*C*; *n* = 188 and 164 cells; *Z* = 4.19; *p* = 2.8 × 10^−5^; 0 vs 1–2, Mann–Whitney *U* test). The same results were also observed from CA1 neurons (Fig. 2*E*; *n* = 62 and 205 cells; *Z* = 4.43; *p* = 9.4 × 10^−6^; 0 vs 1–5, Mann–Whitney *U* test). These results confirm that changes in the overall spike rates of hippocampal neurons during the postrest period reflect the presence of spatial representations formed during prior experiences (Pavlides and Winson, 1989).

### Effects of the number of spatial representations on postexperience SWR-associated reactivation of CA3 and CA1 place cells

Next, we examined how SWR-associated spike patterns of these neurons differed between the prerest and the postrest periods. SWRs were detected from LFP signals during rest periods using conventional procedures (Nishimura et al., 2021; Kuga et al., 2023; Yagi et al., 2023). In our datasets, the rates of SWRs in the CA1, but not CA3, in the postrest period were significantly higher than those in the prerest period (CA3, *n* = 16; *t*_15_ = 0.22; *p* = 0.83; CA1, *n* = 10; *t*_9_ = 3.48; *p* = 0.0070, paired *t* test). For each neuron, averaged SWR-associated spike rates of hippocampal neurons were computed in the pre- and postrest periods (Fig. 2*F*). To compensate for the effects of the postexperience changes in overall spike rates of place cells (Fig. 2*A*–*E*), SWR-associated spike rates were converted to *z*-scored spike rates based on the mean and standard deviation of spike rate changes 100–500 ms before the SWRs in each rest period, yielding *Z*_pre_ and *Z*_post_ (Fig. 2*F*). Scatterplots of *Z*_pre_ and *Z*_post_ were constructed separately for different numbers of rooms in which individual neurons showed place fields (Fig. 2*G*,*I*). CA3 place cells that had place fields in a room showed no significant differences between *Z*_pre_ and *Z*_post_ (Fig. 2*G*; 1, *n* = 101 cells; *Z* = 1.44; *p* = 0.15; 2, *n* = 31 cells; *Z* = 0.65; *p* = 0.52, Wilcoxon signed-rank test), whereas *Z*_post_ was significantly lower than *Z*_pre_ in CA3 neurons that had no place fields in any of the five rooms (*n* = 188 cells; *Z* = 3.12; *p* = 0.0018). To further quantify the strength of postrest SWR-associated reactivation across different neuronal groups, we computed a SWR-induced change ratio for each cell as the ratio of the difference in *Z*_pre_ and *Z*_post_ between the pre- and postrest periods to the sum of the absolute *Z*_pre_ and the absolute *Z*_post_ (Fig. 2*F*, bottom). SWR-induced change ratios of CA3 nonplace cells were significantly lower than those of CA3 place cells that had place fields (Fig. 2*H*; *n* = 188 and 164 cells; *Z* = 3.97; *p* = 7.3 × 10^−5^; 0 vs 1–2, Mann–Whitney *U* test). These results demonstrate that the SWR-associated reactivation of CA3 neurons showing no spatial representation was specifically weakened (Yagi et al., 2023). The significant changes in spike rates in the postrest period were not attributed to alterations in the amplitudes of spike waveforms (e.g., electrode drifting or prominent changes in cell health) between the prerest and postrest periods (Fig. S2*C*). In CA1, there were no significant differences between *Z*_pre_ and *Z*_post_ in place cells that had place fields in any numbers of rooms (Fig. 2*I*; 0, *n* = 62 cells; *Z* = 0.19; *p* = 0.85; 1, *n* = 69 cells; *Z* = 0.019; *p* = 0.99; 2, *n* = 52 cells; *Z* = 0.60; *p* = 0.55; 3, *n* = 32 cells; *Z* = 1.68; *p* = 0.090; 4, *n* = 26 cells; *Z* = 0.040; *p* = 0.97; 5, *n* = 26 cells; *Z* = 1.00; *p* = 0.32, Wilcoxon signed-rank test).

### Effects of the novelty and temporal distance on postexperience changes in overall spike rates of CA3 and CA1 place cells during rest periods

**Figure 3.**
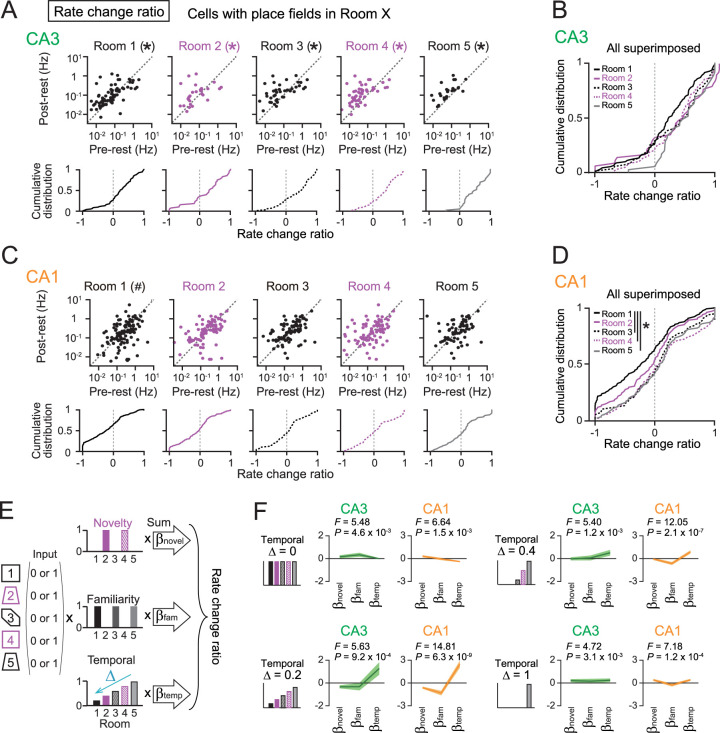
The effects of novelty and temporal distance on postexperience spike rates of place cells during entire rest periods. ***A***, Top, Scatterplots of overall spike rates of CA3 neurons during the postrest period against those during the prerest period, which are separately created for neurons that had place fields in each of the five rooms (Room 1, *n* = 89 cells; *Z* = 3.54; *p = 4.30 × 10^−4^; Room 2, *n* = 38 cells; *Z* = 2.23; *p = 0.026; Room 3, *n* = 52 cells; *Z* = 2.47; *p = 0.013; Room 4, *n* = 59 cells; *Z* = 3.71; *p = 2.1 × 10^−4^; Room 5, *n* = 35 cells; *Z* = 4.65; *p = 3.3 × 10^−6^). *p < 0.05 (postlarger), Wilcoxon signed-rank test. Bottom, The corresponding cumulative distributions of rate change ratios. ***B***, All distributions shown in ***A*** are superimposed. ***C***, Same as ***A*** but for CA1 neurons (Room 1, *n* = 129 cells; *Z* = 3.36; ^#^*p* = 7.8 × 10^−4^; Room 2, *n* = 100 cells; *Z* = 1.45; *p* = 0.15; Room 3, *n* = 96 cells; *Z* = 0.47; *p* = 0.64; Room 4, *n* = 100 cells; *Z* = 0.05; *p* = 0.96; Room 5, *n* = 78 cells; *Z* = 0.75; *p* = 0.45). ^#^*p* < 0.05 (pre larger), Wilcoxon signed-rank test. ***D***, Same as ***B*** but for CA1 neurons (Room 1 vs 3, *Z* = 3.71; *p = 0.0021; Room 1 vs 4, *Z* = 4.38; *p = 1.2 × 10^−4^; Room 1 vs 5, *Z* = 4.00; *p = 6.3 × 10^−4^, Mann–Whitney *U* test followed by Bonferroni’s correction). Results after excluding SWR periods are presented in Figure S4. ***E***, The rate change ratios were linearly regressed against binary-coded spatial representation patterns. Using multiple linear regression, coefficients *β*_novel_, *β*_fam_, and *β*_temp_ were computed. Decay constant Δ represents the decay rate from the previous room. ***F***, *β*_novel_, *β*_fam_, and *β*_temp_ computed from CA3 and CA1 place cells (*n* = 352 and 267 cells from 16 and 10 recording days, respectively). The thick line represents the *β* values, and the shaded area represents the 95% confidence interval.

We then examined how the novelty of each room and its temporal distance from the rest period influenced postexperience neuronal spike rates during entire rest periods (Fig. 3) or rest periods without SWRs (Fig. S4). Similar to Figure 2*A*, rate change ratios were computed but analyzed separately for place cells showing place fields in each room. In CA3, place cells that had place fields in all rooms showed significantly higher spike rates in the postrest period than those in the prerest period (Fig. 3*A*; Room 1, *n* = 89 cells; *Z* = 3.54; *p* = 4.30 × 10^−4^; Room 2, *n* = 38 cells; *Z* = 2.23; *p* = 0.026; Room 3, *n* = 52 cells; *Z* = 2.47; *p* = 0.013; Room 4, *n* = 59 cells; *Z* = 3.71; *p* = 2.1 × 10^−4^; Room 5, *n* = 35 cells; *Z* = 4.65; *p* = 3.3 × 10^−6^, Wilcoxon signed-rank test), confirming increases in overall spike rates of subsets of CA3 place cells during the postrest period. Consistent significant results were obtained from rest periods excluding SWRs (Fig. S4*A*). In CA1, no significant differences in overall spike rates of place cells showing place fields in Rooms 2–5 were observed (Fig. 3*C*; Room 2, *n* = 100 cells; *Z* = 1.45; *p* = 0.15; Room 3, *n* = 96 cells; *Z* = 0.47; *p* = 0.64; Room 4, *n* = 100 cells; *Z* = 0.05; *p* = 0.96; Room 5, *n* = 78 cells; *Z* = 0.75; *p* = 0.45, Wilcoxon signed-rank test). On the other hand, place cells that had place fields in Room 1 showed significantly lower spike rates in the postrest period than those in the prerest period (Room 1, *n* = 129 cells; *Z* = 3.36; *p* = 7.8 × 10^−4^). Consistent significant results were obtained from rest periods excluding SWRs (Fig. S4*C*). These results suggest that overall spike rates of CA1 place cells during the postrest period, irrespective of SWRs, decrease when these cells have encoded information related to temporally distant events and subsequently experienced multiple environments.

To more directly estimate the effects of the novelty and temporal distance on the rate change ratios, a multiple linear regression approach was employed in which the rate change ratios of individual neurons (dependent variable) were regressed from three vectors (Fig. 3*E*). This regression analysis was applied to pooled data from all rats (*n* = 352 and 267 cells from CA3 and CA1, respectively). The first predictor vector was the product of binary indices indicating place field presence (1) or absence (0) and a constant binary variable (1 or 0) indicating “novelty” in each room. The second predictor vector was similarly constructed but with a binary variable (1 or 0) indicating “familiarity.” The third predictor vector was the multiplication of the same binary indices and a constant variable composed of a time function assuming that neuronal activity underlying memory decays over time (Tsao et al., 2018; Candia et al., 2019). Since the decay time constant in each room was unknown, we tested various decay constants Δ to model different attenuation rates, with larger Δ values indicating stronger weakening of effects from earlier rooms (e.g., Δ = 0.2, vector [0.2, 0.4, 0.6, 0.8, 1]; Δ = 0.4, vector [0, 0, 0.2, 0.6, 1]). The regression yielded the coefficients *β*_novel_, *β*_fam_, and *β*_temp_, representing the effects of novelty, familiarity, and temporal decay, respectively. We performed regression analysis under four conditions: Δ = 0, 0.2, 0.4, and 1 (Fig. 3*F*). In both CA3 and CA1, the *F* values and *p* values in all conditions were statistically significant (*p* < 0.05), confirming the validity of this model. Both CA3 and CA1 showed similar trends: when Δ = 0.2, *β*_temp_ was notably the largest and remained clearly positive even when considering the 95% confidence interval. The effect was less pronounced under other conditions. These results suggest that Δ = 0.2 best captures the time-dependent temporal effect. In contrast, across all Δ values including the condition of Δ = 0.2, neither *β*_novel_ nor *β*_fam_ showed significant deviation from zero, even when considering their 95% confidence intervals. Taken together, these results suggest that postexperience changes in spike rates of both CA3 and CA1 place cells during entire rest periods are primarily determined by the temporal distance from when the cells had place fields, particularly under conditions where the effect gradually decays across the five rooms (Δ = 0.2) rather than by the novelty or familiarity of experiences.

### Effects of the novelty and temporal distance on postexperience SWR-associated reactivation of CA3 and CA1 place cells

**Figure 4.**
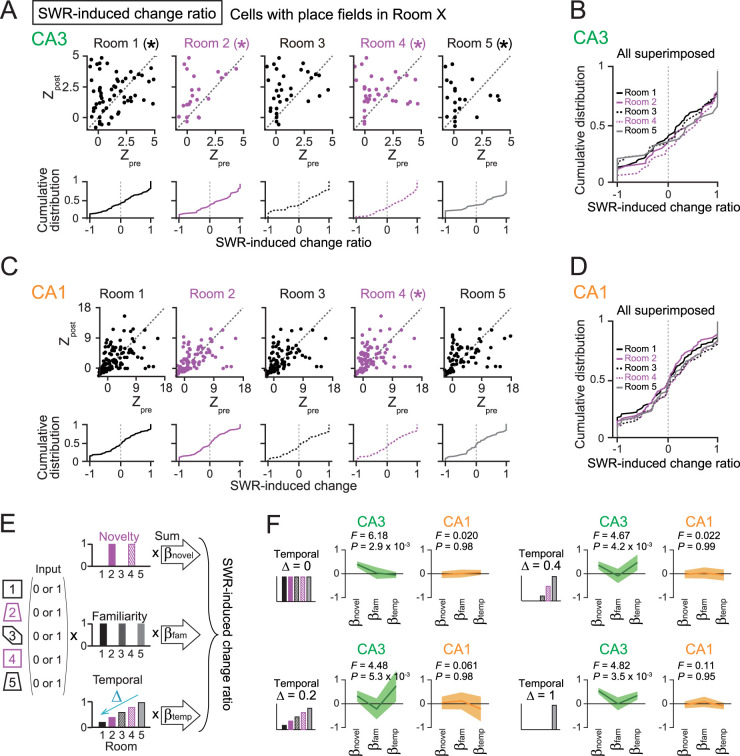
The effects of novelty and temporal distance on postexperience SWR-induced reactivation of place cells. ***A***, Top, Scatterplots of SWR-associated spike rates of CA3 neurons during the postrest period against those during the prerest period, separately created for neurons that had place fields in each of the five rooms (Room 1, *n* = 89 cells; *Z* = 2.64; *p = 0.0082; Room 2, *n* = 38 cells; *Z* = 2.23; *p = 0.026; Room 3, *n* = 52 cells; *Z* = 1.57; *p* = 0.096; Room 4, *n* = 59 cells; *Z* = 3.94; *p = 8.0 × 10^−5^; Room 5, *n* = 35 cells; *Z* = 2.47; *p = 0.014). *p < 0.05 (postlarger), Wilcoxon signed-rank test. Bottom, The corresponding cumulative distributions of SWR-induced change ratios. Relationship between SWR-induced change ratios and spatial information are presented in Figure S5. ***B***, All distributions shown in ***A*** are superimposed. ***C***, Same as ***A*** but for CA1 neurons (Room 1, *n* = 129 cells; *Z* = 0.72; *p* = 0.47; Room 2, *n* = 100 cells; *Z* = 0.18; *p* = 0.86; Room 3, *n* = 96 cells; *Z* = 1.73; *p* = 0.085; Room 4, *n* = 100 cells; *Z* = 2.11; *p = 0.035; Room 5, *n* = 78 cells; *Z* = 1.09; *p* = 0.28, Wilcoxon signed-rank test). ***D***, Same as ***B*** but for CA1 neurons. ***E***, The SWR-induced change ratios were linearly regressed against binary-coded spatial representation patterns. Using multiple linear regression, coefficients *β*_novel_, *β*_fam_, and *β*_temp_ were computed. Decay constant Δ represents the decay rate from the previous room. ***F***, *β*_novel_, *β*_fam_, and *β*_temp_ computed from CA3 and CA1 place cells (*n* = 352 and 267 cells from 16 and 10 recording days, respectively). The thick line represents the *β* values, and the shaded area represents the 95% confidence interval.

We then examined how the novelty and temporal distance explain changes in postexperience SWR-induced spike patterns. Same as Figure 2*F*, the effects of the changes in the overall spike rates of place cells during rest periods were compensated by computing *Z*_pre_, *Z*_post_, and SWR-induced change ratios. In both CA3 and CA1, SWR-induced change ratios were not significantly correlated with spatial information density computed in each room (Fig. S5). Plotting *Z*_pre_ and *Z*_post_ separately for each room revealed significantly higher *Z*_post_ than *Z*_pre_ in CA3 place cells that had place fields in each room, except Room 3 (Fig. 4*A*, top; Room 1, *n* = 89 cells; *Z* = 2.64; *p* = 0.0082; Room 2, *n* = 38 cells; *Z* = 2.23; *p* = 0.026; Room 3, *n* = 52 cells; *Z* = 1.57; *p* = 0.096; Room 4, *n* = 59 cells; *Z* = 3.94; *p* = 8.0 × 10^−5^; Room 5, *n* = 35 cells; *Z* = 2.47; *p* = 0.014, Wilcoxon signed-rank test). In CA1, place cells that had place fields in Room 4 showed significantly higher *Z*_post_ than *Z*_pre_ (Fig. 4*C*, top; Room 1, *n* = 129 cells; *Z* = 0.72; *p* = 0.47; Room 2, *n* = 100 cells; *Z* = 0.18; *p* = 0.86; Room 3, *n* = 96 cells; *Z* = 1.73; *p* = 0.085; Room 4, *n* = 100 cells; *Z* = 2.11; *p* = 0.035; Room 5, *n* = 78 cells; *Z* = 1.09; *p* = 0.28, Wilcoxon signed-rank test).

To further estimate the effects of the novelty and temporal distance on the SWR-induced change ratios, a multiple linear regression approach was employed (Fig. 4*E*), similar to Figure 3*E*. The regression analysis was also tested under four conditions: Δ = 0, 0.2, 0.4, and 1 (Fig. 4*F*). In CA3, the *F* values and *p* values in all conditions were statistically significant (*p* < 0.05), confirming the validity of this model. When Δ = 0.2 or 0.4, *β*_temp_ was positive, with the lower bound of the 95% confidence interval remaining above zero. This result suggests that temporal decay at this degree may influence the SWR-induced change ratio in CA3. Additionally, across all Δ conditions including Δ = 0 (assuming no temporal effect), *β*_novel_ was positive even when considering the lower bound of the 95% confidence interval, whereas no such positive effect was detected for *β*_fam_. In CA1, the *F* values and *p* values in all conditions were not statistically significant (*p* > 0.05), indicating that the model did not fit the data well. All *β* coefficients were not clearly different from zero, meaning that SWR-induced change ratios in CA1 cells were not clearly related to temporal, novelty, or familiarity effects. Taken all, these results suggest that increases in postexperience SWR-induced reactivation of CA3 place cells are primarily determined by environmental novelty. The temporal distance from when the cells had place fields also contributes to their postexperience SWR-induced reactivation.

### Coordinated spikes of hippocampal cell ensembles during postrest periods

**Figure 5.**
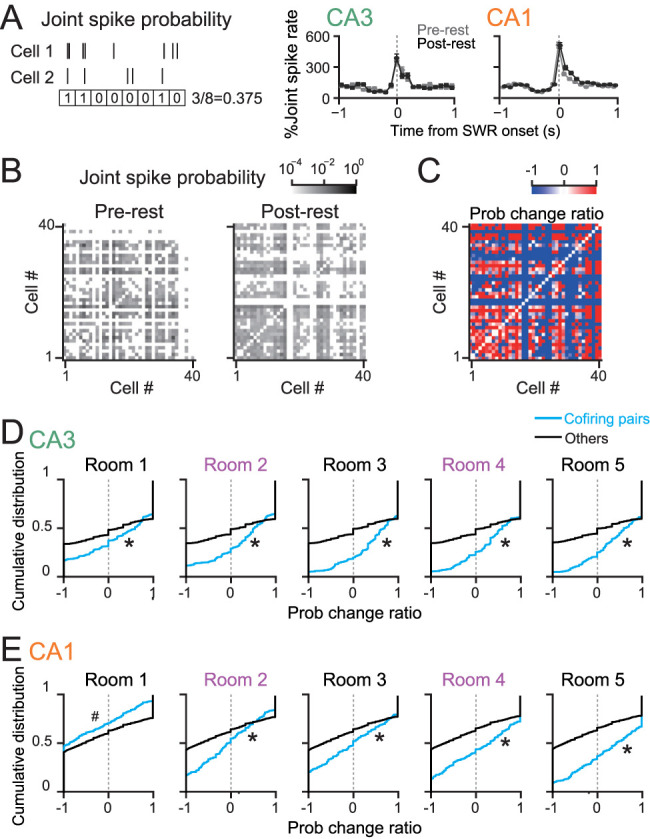
Changes in joint spikes of neuron pairs during the postexperience rest period. ***A***, Left, Schematic illustration to compute joint firing probabilities during a rest period for each neuron pair. Right, SWR-triggered changes in joint spike rates in the pre- and postrest periods, averaged over all neuron pairs (CA3, 259 neuron pairs; CA1, 646 neuron pairs). Data are presented as the mean ± SEM. ***B***, Probability matrices for all pairs of neurons computed from the pre- (left) and postrest (right) periods in a rat. ***C***, A matrix showing probability change ratios computed from the two probability matrices in ***B***. ***D***, Comparison of distributions of probability change ratios between CA3 neuron pairs showing significant cofiring_room_ in a room (cyan) and the other pairs (black), separately created for each room in which cofiring_room_ of the neuron pairs was computed (Room 1, *n* = 304 and 9,452 cell pairs; *Z* = 2.72; *p = 0.0066; Room 2, *n* = 404 and 9,352 cell pairs; *Z* = 4.47; *p = 7.6 × 10^−6^; Room 3, *n* = 328 and 9,428 cell pairs; *Z* = 6.01; *p = 1.8 × 10^−9^; Room 4, *n* = 332 and 9,424 cell pairs; *Z* = 5.75; *p = 9.1 × 10^−9^; Room 5, *n* = 446 and 9,310 cell pairs; *Z* = 6.92; *p = 4.4 × 10^−12^; Mann–Whitney *U* test). ***E***, Same as ***D*** but for CA1 neuron pairs (Room 1, *n* = 598 and 7,524 cell pairs; *Z* = 4.89; ^#^*p* = 1.9 × 10^−6^; Room 2, *n* = 480 and 7,642 cell pairs; *Z* = 6.99; *p = 2.8 × 10^−12^; Room 3, *n* = 480 and 7,642 cell pairs; *Z* = 6.79; *p = 1.1 × 10^−11^; Room 4, *n* = 532 and 7,590 cell pairs; *Z* = 11.59; *p = 4.6 × 10^−31^; Room 5, *n* = 520 and 7,602 cell pairs; *Z* = 14.90; *p = 3.3 × 10^−50^, Mann–Whitney *U* test).

Finally, these postrest spike patterns were examined at the cell ensemble level. A binary vector was constructed from each cell with entries of 1 (with spikes) or 0 (no spikes) in each time bin (100 ms), and a joint spike probability of two vectors from each neuron pairs was computed in each pre- and postrest period (Fig. 5*A*, left). Joint spike rates markedly increased during SWR generation, verifying the temporal alignment of synchronous spikes with SWRs (Fig. 5*A*, right). For each neuronal pair, a change in joint spike probabilities from the pre- to the postrest period (Fig. 5*B*, left and right) was quantified by a probability change ratio that represents the difference in two population proportions (Fig. 5*C*). In addition to these parameters from the rest periods, to define which neuronal pairs showed coordinated spike patterns during experiencing the five rooms, cofiring_room_ was computed from each neuronal pair in each room. Cofiring pairs in each room were defined as neuron pairs with cofiring_room_ exceeding 0.03. For cofiring pairs defined in each room, probability change ratios were compared with the other neuron pairs. In CA3, cofiring pairs defined in individual rooms showed significantly higher probability change ratios than the other cell pairs (Fig. 5*D*; Room 1, *n* = 304 and 9,452 cell pairs; *Z* = 2.72; *p* = 0.0066; Room 2, *n* = 404 and 9,352 cell pairs; *Z* = 4.47; *p* = 7.6 × 10^−6^; Room 3, *n* = 328 and 9,428 cell pairs; *Z* = 6.01; *p* = 1.8 × 10^−9^; Room 4, *n* = 332 and 9,424 cell pairs; *Z* = 5.75; *p* = 9.1 × 10^−9^; Room 5, *n* = 446 and 9,310 cell pairs; *Z* = 6.92; *p* = 4.4 × 10^−12^, Mann–Whitney *U* test). These results demonstrate that postexperience increases in coordinated spiking occur in CA3 neuronal ensembles that encoded each experience within multiple experiences, regardless of their novelty or familiarity.

In CA1, cofiring pairs defined in individual rooms showed significantly higher probability change ratios than the other cell pairs (Room 2, *n* = 480 and 7,642 cell pairs; *Z* = 6.99; *p* = 2.8 × 10^−12^; Room 3, *n* = 480 and 7,642 cell pairs; *Z* = 6.79; *p* = 1.1 × 10^−11^; Room 4, *n* = 532 and 7,590 cell pairs; *Z* = 11.59; *p* = 4.6 × 10^−31^; Room 5, *n* = 520 and 7,602 cell pairs; *Z* = 14.90; *p* = 3.3 × 10^−50^, Mann–Whitney *U* test), leading to the similar suggestion to CA3 neurons. On the other hand, cofiring pairs defined in Room 1 showed significantly lower probability change ratios than the other cell pairs (Room 1, *n* = 598 and 7,524 cell pairs; *Z* = 4.89; *p* = 1.9 × 10^−6^, Mann–Whitney *U* test). This result suggests that postexperience coordinated spikes are decreased in CA1 neurons that have encoded information related to temporally distant events and subsequently experienced multiple events. While these coordinated spikes may represent spike sequences of replays of behavior (Dupret et al., 2010; Jadhav et al., 2012; Silva et al., 2015; Roux et al., 2017; Igata et al., 2021), owing to the limitations of the spike samples, our spike analysis did not analyze the fine orders of spikes at millisecond timescales.

## Discussion

**Figure 6.**
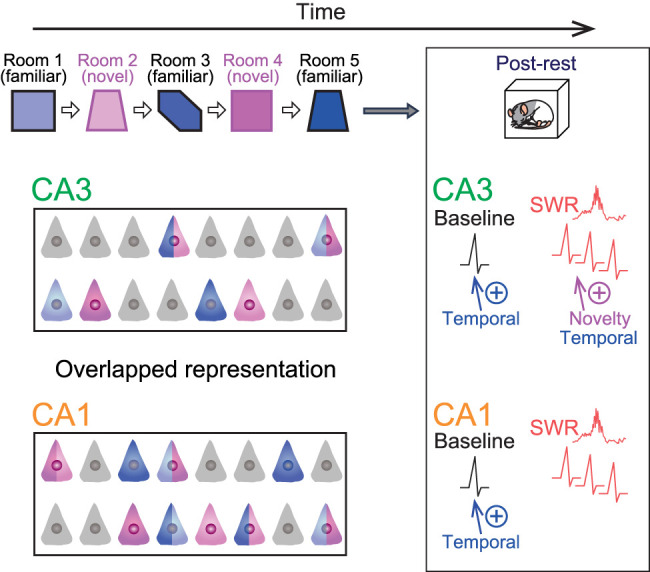
Schematic illustration summarizing the results. Left, Spatial representations in multiple rooms. Place cells identified from the five rooms are labeled in corresponding colors. The cells that had place fields in multiple rooms are represented by two colors. Nonplace cells are colored in gray. Right, During postrest periods, changes in baseline spike rates of CA3 and CA1 neurons are mainly affected by the temporal distance from when the neurons showed place fields. In addition, increases in SWR-induced spikes of CA3 place cells are affected by the novelty and temporal distance.

We analyzed spike patterns of hippocampal place cells in the CA3 and CA1 areas as rats sequentially foraged in familiar and novel rooms, followed by a rest period. Across multiple environments, both CA3 and CA1 place cells showed overlapping spatial representations (Leutgeb et al., 2004, 2007; Alme et al., 2014; Fig. 6). During entire postrest periods, both CA3 and CA1 showed changes in spike rates that depended on the temporal distance from when the cells had place fields. On the other hand, CA3 place cells showed increased SWR-associated reactivation, with the strong effects observed for cells that had encoded novel environments, in accordance with a previous study (Hwaun and Colgin, 2019). CA3 neuron pairs with place fields in all rooms showed significant increases in coordinated spikes in the postrest period. CA1 neuron pairs with place fields in Rooms 2–5 showed significant increases in coordinated spikes in the postrest period.

The presence of place cell ensembles that consistently encode the same places across different environments suggests that these cells form overlapping spatial maps to store multiple spatial memories. This encoding feature can efficiently reduce the sparsity and variability in the spatial representation patterns of neuronal ensembles in multiple environments. Possible mechanisms underlying these ensemble encoding patterns are common synaptic inputs into the CA1 neuronal subpopulation from the CA3 area and CA3 recurrent networks (Leutgeb et al., 2004, 2007; Takahashi et al., 2010; Alme et al., 2014; Kong et al., 2024) and/or the temporoammonic pathway from the entorhinal cortex processing multimodal spatial information (Witter et al., 2017). In addition, the interconnections of pyramidal cells and feed-forward and feedback networks that emit common inhibitory inputs within the circuit may be effective in sustaining specific place cell ensembles that encode similar information.

Our results suggest that information related to novelty is mainly processed by postexperience SWR-associated neuronal reactivation in the CA3 and CA1 hippocampal circuit. These observations are consistent with those observed in CA1 neurons that encode information related to reward (Dupret et al., 2010). Taken together, novelty- and reward-related information serves as a stronger driver of coordinated reactivation in subsequent rest periods, irrespective of temporal distance-related information, to consolidate these memories within the hippocampal circuit (Wagatsuma et al., 2018; Hwaun and Colgin, 2019; Ecker et al., 2022). Possible mechanisms underlying the novelty-biased information processing include NMDA receptor-dependent synaptic plasticity (Nakazawa et al., 2003) and the abundant transmission of neuromodulatory signals from the locus ceruleus (Takeuchi et al., 2016; Wagatsuma et al., 2018). We found that the strength of postexperience CA3 and CA1 neuronal activation is primarily determined by temporal distance, whereas the novelty of the environment had little influence. Specifically, neurons that had no place field or encoded the first environment exhibited decreased activation. These results suggest that distant memory is interfered with or overwritten by recent memory during activation of entire rest periods. Overall, the direction of experience-induced changes in the strength of neuronal activation may be counterbalanced throughout the hippocampal circuit. The mechanisms underlying elapsed time-dependent activation can be attributed to plastic changes or fluctuations in neuronal codes that accumulate over time and experience. The temporal information-related activation features in hippocampal neurons during subsequent experiences resemble their spatial encoding characteristics, where CA1 neurons exhibit time-varying spatial encoding even within the same environments (Mankin et al., 2012; Sheintuch et al., 2023).

In conclusion, the CA3 and CA1 circuits ensure offline SWR-induced reactivation of novelty-related information rather than temporal distance-related information. Conversely, time-related information is represented by the baseline spike rates of these neurons during entire rest periods, independent of SWR events.

## Data Availability

- The original data and codes are available from the corresponding author upon request.
- Any additional information required to reanalyze the data reported in this paper is available from corresponding author upon request.
